# Comparison of Four Screening Markers [(C16 + C18:1)/C2, C14/C3, C12/C0, and C12/C2] for Carnitine Palmitoyltransferase II Deficiency in the Nationwide Newborn Screening Program in Japan

**DOI:** 10.3390/ijns12020036

**Published:** 2026-05-15

**Authors:** Go Tajima, Nobuyuki Ishige, Junji Hanai, Keiko Konomura

**Affiliations:** 1 Division of Neonatal Screening, Research Institute, National Center for Child Health and Development, 2-10-1 Okura, Setagaya 157-8535, Japan; 2 Division of Newborn Screening, Tokyo Health Service Association, 1-2-59 Ichiga-Sadohara, Shinjuku 162-8460, Japan; 3 Hokkaido Pharmaceutical Association Public Health Examination Center, 8-6-6 Hiragishi 1-jo, Toyohira 062-0931, Japan; 4 Center for Outcomes Research and Economic Evaluation for Health (C2H), National Institute of Public Health, 2-3-6 Minami, Wako 351-0197, Japan

**Keywords:** carnitine palmitoyltransferase II, CPT II, newborn screening, false positive, dodecanoylcarnitine (C12)

## Abstract

False-positive results are known to occur frequently in newborn screening (NBS) for carnitine palmitoyltransferase II (CPT II) deficiency, highlighting the need to identify appropriate screening markers. The present study aimed to compare the performance of two markers currently used in NBS for CPT II deficiency, (C16 + C18:1)/C2 and C14/C3, with two promising alternative markers, C12/C0 and C12/C2. We analyzed non-patient data from the 2023 fiscal year NBS program together with patient data for CPT II deficiency and very-long-chain acyl-CoA dehydrogenase deficiency derived from previously reported case series. Marker performance was assessed using precision–recall curves, including an analysis in which patients with the myopathic form of CPT II deficiency who passed NBS using (C16 + C18:1)/C2 and C14/C3 were reclassified as true positives. The area under the precision–recall curve values for C12/C2 and C14/C3 were 0.711 (95% confidence interval, 0.492–0.923) and 0.569 (0.341–0.779), respectively, indicating superior performance compared with the other markers. When cases with the myopathic form of CPT II deficiency were included as true positives, the performance of all markers decreased markedly. Although some patients with the myopathic form are still likely to be missed, C12/C2 appears to be an effective marker for reducing false-positive results.

## 1. Introduction

Carnitine palmitoyltransferase II (CPT II) deficiency is a fatty acid oxidation disorder caused by reduced activity or deficiency of CPT II, an enzyme located in the mitochondrial inner membrane. CPT II deficiency is clinically classified into three phenotypes: (1) a lethal neonatal form associated with cardiomyopathy; (2) a severe infantile form (hereafter referred to as the “hypoglycemic form”), characterized by hypoglycemia, Reye-like encephalopathy, and in the severe cases, cardiopulmonary arrest during infancy and early childhood; and (3) an adult-onset form (hereafter referred to as the “myopathic form”) presenting with recurrent rhabdomyolysis in adolescence or later, although myopathic symptoms can appear even in early childhood.

The epidemiology of CPT II deficiency shows geographic variation. In populations of European descent, the myopathic form predominates and is commonly associated with the c.338C>T (p.S113L) variant [1]. In contrast, the hypoglycemic form is more prevalent in Japan, where variants such as c.1148T>A (p.F383Y) and c.520G>A (p.E174K) have been frequently identified in patients presenting with hypoglycemia, Reye-like encephalopathy, and sudden death [2,3].

Tandem mass spectrometry (MS/MS)-based newborn screening (NBS) was introduced in Japan in 1997 as a pilot study, in which CPT II deficiency was screened using C16 and C18:1 as markers. Subsequently, the effectiveness of (C16 + C18:1)/C2 and C14/C3 as improved screening markers were reported [4], and CPT II deficiency was added to the official nationwide NBS panel in 2018 [3]. The current screening strategy using (C16 + C18:1)/C2 and C14/C3 effectively prevents the catastrophic onset of the hypoglycemic form of the disease, although a small number of myopathic cases with a low risk of severe clinical outcomes have been missed by the current NBS [5]. Nevertheless, these markers remain associated with a high false-positive rate, which increases the burden on children and families, screening laboratories, and clinical management.

Furthermore, additional challenges arise in the management of screen-positive cases. Among individuals who tested positive for CPT II deficiency in NBS but showed normal results on enzymatic assays and serum acylcarnitine analysis, the c.1055T>G (p.F352C) variant has been detected in a considerable number of cases by sequencing of *CPT2*. This allele, known as a thermolabile variant, has been reported as an important risk factor for acute infectious encephalopathy (AIE) in Japanese children [6,7]. However, data from the Tohoku Medical Megabank, including approximately 3,800 Japanese individuals, indicate that the allele frequency of c.1055G is 0.18274 [8]. Although this frequency appears too high for the variant to act as a sole cause of AIE, its detection in NBS-positive cases complicates clinical decision-making regarding their management. These issues highlight the need for improved screening markers that reduce the false-positive rate without compromising sensitivity.

To explore markers with better performance, we performed receiver operating characteristic (ROC) analysis of dried blood spot (DBS) acylcarnitine profiles from 76 NBS-positive cases with (C16 + C18:1)/C2 and C14/C3, of whom 21 were true patients. Five symptomatic patients who had been missed by NBS using these markers were also included in the analysis. As a result, C12/C0 was revealed as the most sensitive marker [5]. After the publication, we tried to check its performance on NBS data of 18,455 newborns in Hiroshima area tested in 2022, which resulted in unexpectedly high positive rate. We applied C12/C2, which had shown slightly lower sensitivity but higher specificity than C12/C0 [5], to the same data set. As a result, it successfully detected a confirmed patient born in the year, and only two other newborns were judged as positive (unpublished data). It is necessary to validate the performance of these markers using highly representative data.

In the current study, we validated the performance of these two markers by applying them to nationwide NBS data in Japan.

## 2. Materials and Methods

### 2.1. Study Population

This study was a retrospective analysis of DBS samples collected from newborns screened as part of Japan’s nationwide NBS program. Anonymized data for 683,391 non-patient newborns were collected from the screening records for the 2023 fiscal year across 34 testing laboratories in Japan. Data from 26 confirmed CPT II deficiency cases diagnosed between 2004 and 2023, including 5 cases with the myopathic form that were negative with the current NBS, were obtained from previous research [5]. These facilities collectively covered approximately 95.5% of the 715,728 live births recorded in Japan in fiscal year 2023 [9]. As very-long-chain acyl-CoA dehydrogenase (VLCAD) deficiency cases can occasionally appear as false positives for CPT II deficiency markers, we included 95 VLCAD deficiency cases identified through NBS in our analysis. Their NBS data were described in a previous study [10]. These data were derived from patients who were identified as positive through an NBS program and subsequently diagnosed with the disease following additional diagnostic testing. All cases had results for various acylcarnitines and their ratios obtained from the initial NBS.

### 2.2. Screening Test for CPT II Deficiency

DBSs for NBS are generally collected on postnatal days 4 or 5 and sent to testing laboratories across Japan for analysis, in accordance with the official protocol used since the start of NBS for amino acid disorders in 1977, where a uniform filter paper (Advantec Toyo Kaisha, Tokyo, Japan) has been used. Blood samples were analyzed by MS/MS following the protocol described in a previous report [11] with some modification. With respect to MS/MS and sample preparation kits, including stable-isotope-labeled internal standards, products from several manufacturers are used in various combinations. However, inter-laboratory variation is monitored and maintained within acceptable limits by the Quality Control Committee of the Japanese Society for Neonatal Screening.

Indices for CPT II deficiency were as follows: (1) during the pilot study from 2004 to 2010, C16 ≥ 6.3 nmol/mL and C18:1 ≥ 3.6 nmol/mL, both cutoff values corresponding to the mean + 4 SD when they were set [4], (2) during the pilot study from 2011 to 2017, (C16 + C18:1)/C2 ≥ 0.62 (99.9th percentile in healthy control newborns) and C16 ≥ 3.0 nmol/mL (79.5th percentile) [4], (3) after the start of nationwide NBS in April 2018, (C16 + C18:1)/C2 and C14/C3 with both cutoff values set at the 99.9th percentile [3]. Nationwide NBS testing is performed by 35 regional laboratories, where the cutoff values used (mean ± SD) were 0.432 ± 0.073 for (C16 + C18:1)/C2 and 0.386 ± 0.077 for C14/C3, respectively.

As indices for VLCAD deficiency, C14:1 ≥ 0.4 nmol/mL and C14:1/C2 ≥ 0.013 were used during the pilot study from 1997 to 2012; both cutoff values corresponded to the 99.5th percentile of values in healthy control newborns. In the nationwide NBS since 2013, the mean cutoff values used in 35 regional laboratories were 0.34 ± 0.06 nmol/mL for C14:1 and 0.013 ± 0.004 for C14:1/C2 [10].

When a screening test yields a positive result, the findings are forwarded from screening laboratories to medical centers, where further confirmatory testing is performed.

### 2.3. Statistical Analysis

Descriptive statistics for continuous variables were expressed as the mean, standard deviation, median, and interquartile range (IQR). We evaluated the performance of each screening marker both with and without inclusion of cases with the myopathic form of CPT II deficiency as true-positive cases. To assess the performance of the screening markers, the area under the receiver operating characteristic curve (ROC-AUC) and the corresponding 95% confidence interval (CI) were calculated. The optimal cutoff values for each marker were determined using the Youden index derived from the ROC curve, and sensitivity, specificity, positive predictive value (PPV), and negative predictive value (NPV) were calculated based on these cutoff values. Because CPT II deficiency is an extremely rare disorder, resulting in a highly imbalanced dataset between positive and negative cases, the area under the precision–recall curve (PR-AUC) was adopted to evaluate performance for the positive class. The 95% CIs for the PR-AUC were estimated using the non-parametric percentile bootstrap method, with 10,000 replicates generated by resampling the original dataset with replacement. Scatter plots were constructed using each CPT II marker together with the VLCAD marker C14:1 to visually examine the relationships among the data. All analyses were performed using RStudio version 2025.09.0 (Posit Software, PBC, Boston, MA, USA) with R version 4.4.0. The pROC package in R was used to generate ROC curves, and the PRROC package was used to generate PR curves.

## 3. Results

### 3.1. Characteristics of Screening Markers

The characteristics of the screening markers for CPT II deficiency cases and non-patients are summarized in Table 1. CPT II deficiency cases tended to show higher values of the screening markers, except for the five with myopathic form.

### 3.2. Performance of the Markers Excluding Myopathic Form

**Figure 1:**
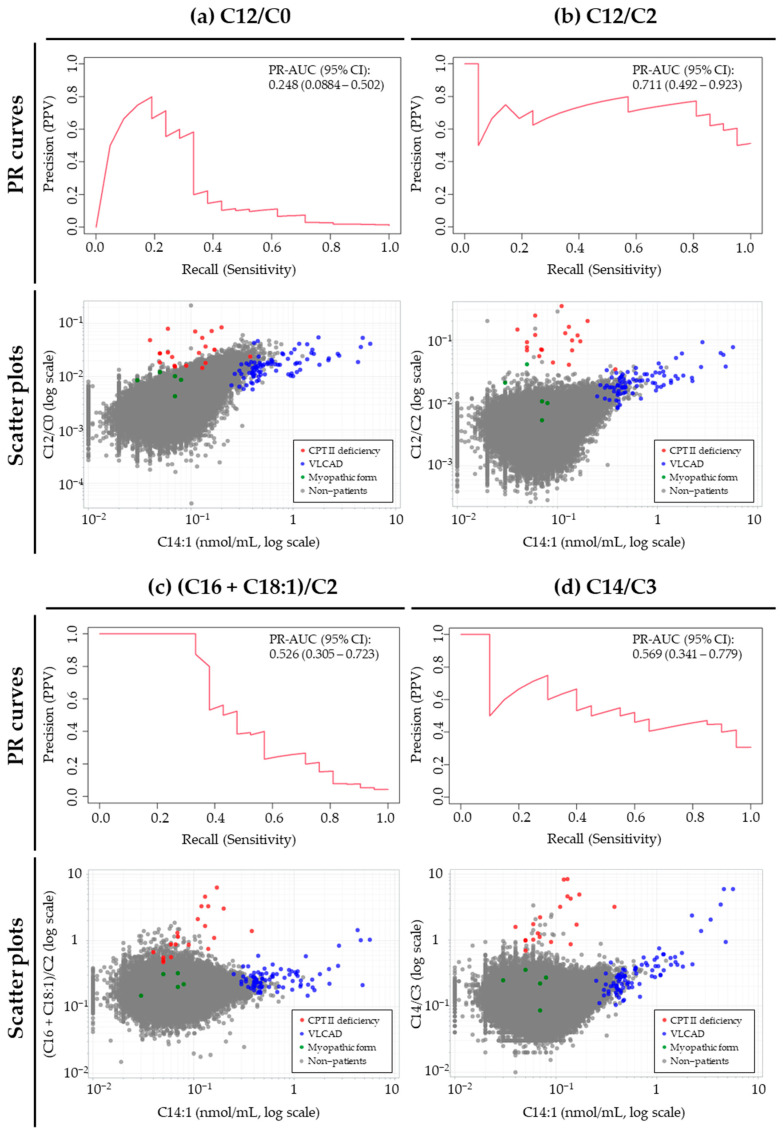
Precision–Recall curves and scatter plots for the four screening markers for CPT II deficiency. 95% CI, 95% confidence interval; PR-AUC, area under the precision–recall curve; PR curve, precision–recall curve; VLCAD, very-long-chain acyl-CoA dehydrogenase. The total numbers of samples analyzed for C12/C0, C12/C2, (C16 + C18:1)/C2, and C14/C3 were 668,035, 668,035, 600,116, and 619,375, respectively, and the numbers of patients with CPT II deficiency were 21, 21, 21, and 20, respectively. Data are reported to three significant digits.

The performance of the screening markers for CPT II deficiency without the myopathic form is summarized in Table 2 (ROC analysis), while the corresponding PR curves and scatter plots are shown in Figure 1. The ROC-AUC values were nearly 1.00 for all screening markers. According to the cutoff values determined by the Youden Index, sensitivity was 100% for all markers, and specificity was greater than 99.8%. C12/C2 and C14/C3 showed higher PPVs (51.2% and 30.8%, respectively) and tended to produce fewer false positives than the other markers. The numbers of false positives for C12/C2, C14/C3, (C16 + C18:1)/C2 and C12/C0 were 20, 45, 458, and 1,312, respectively. C12/C2 showed the highest PR-AUC (0.711; 95% CI, 0.492–0.923), followed by C14/C3 (0.569; 95% CI, 0.341–0.779), (C16 + C18:1)/C2 (0.526; 95% CI, 0.305–0.723) and C12/C0 (0.248; 95% CI, 0.0884–0.502), suggesting that C12/C2 had the highest discriminative performance among the evaluated markers. The scatter plots show that some VLCAD cases exhibit higher values within the range of each CPT II marker, although they tended to be distinguishable by their high C14:1 level.

### 3.3. Performance of the Markers Including Myopathic Form

**Figure 2:**
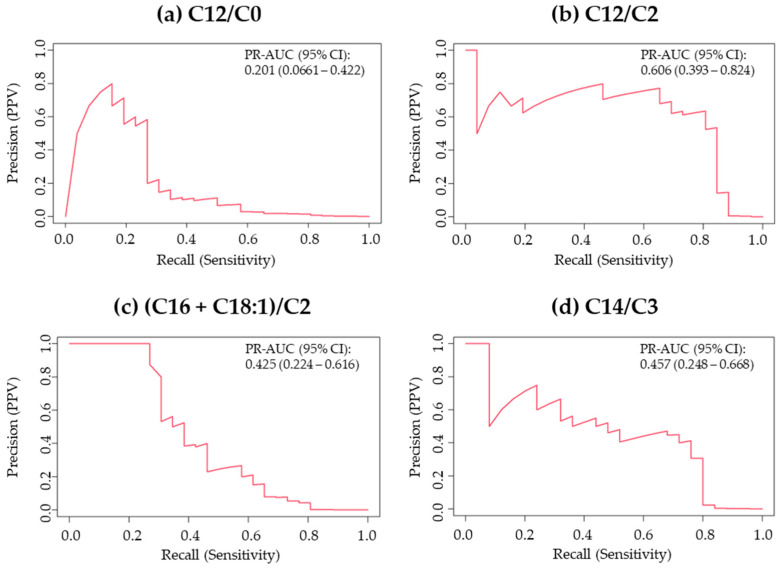
Precision–Recall curves for the four screening markers for CPT II deficiency including myopathic form. 95% CI, 95% confidence interval; PPV, positive predictive value; PR-AUC, area under the precision–recall curve. The total numbers of samples analyzed for C12/C0, C12/C2, (C16 + C18:1)/C2, and C14/C3 were 668,035, 668,035, 600,116, and 619,375, respectively, and the numbers of patients with CPT II deficiency were 26, 26, 26, and 25, respectively. Data are reported to three significant digits.

When patients with the NBS-negative myopathic form were included as true positive, the performance of all markers declined substantially (see Supplementary Figure S1). The ROC-AUC, which had been nearly 100% when myopathic cases were excluded, decreased and resulted in the occurrence of false negatives at the optimal cutoff values. Furthermore, the PR curves also showed a marked decline in PPV at sensitivity levels above 80%, indicating an increasing number of false positives (Figure 2).

## 4. Discussion

In this study, we compared the performance of four screening markers for CPT II deficiency using data from case reports and nationwide NBS. Although the small number of CPT II deficiency cases introduces some uncertainty into the results, C12/C2 may represent the best overall option for reducing false-positive results.

We evaluated the performance of the screening markers using PR curves, given that the dataset was highly imbalanced and contained only a very small number of patients. C12/C2 demonstrated the highest PR-AUC, followed by C14/C3. However, the 95% CIs for the PR-AUC values were wide and overlapped across all markers, suggesting considerable uncertainty in the estimates. Importantly, CPT II deficiency is an extremely rare disorder, and the present analysis was based on the largest dataset currently available in Japan, accumulated over 20 years. Continued longitudinal data collection will be essential to improve the reliability of these screening markers.

C12/C2 and C14/C3 maintained relatively high PPV values across the range of the sensitivities observed in the PR curves. Since PPV is affected by disease prevalence, it is common for PPV to become extremely low in highly imbalanced datasets with very rare conditions, such as CPT II deficiency. However, the observed PPVs for C12/C2 and C14/C3 were relatively high at 51.2% and 30.8%, respectively, indicating a small number of false positives. On the other hand, the PR curves for (C16 + C18:1)/C2 and C12/C0 showed a marked decrease in PPV as sensitivity increased, indicating that maintaining high sensitivity with these markers results in a large number of false positives. Similar findings for (C16 + C18:1)/C2 have been reported previously [3]. As for C12/C0, it has been reported to have a high ability to distinguish affected patients among NBS-positive infants [5]; however, our results suggest that this marker may produce a substantial number of false positives. Previous studies used data only from NBS-positive subjects who underwent confirmatory testing, and thus the distribution of marker values among the vast majority of newborns who passed NBS was not evaluated. These patterns can also be visually observed in the scatter plots shown in Figure 1.

The ability to distinguish cases with myopathic-form symptoms is limited across all markers. The distribution of marker values in the five myopathic cases that developed symptoms after they passed NBS largely overlapped with those observed in non-patient newborns. Consequently, the performance of all markers declined substantially when these cases were included as true-positive cases. Considering that the onset of myopathic symptoms without hypoglycemia does not lead to severe neurological sequelae or fatal outcomes, and that they are difficult to completely prevent even after the diagnosis, priority should be given to reducing false-positive results, even at the cost of missing some patients with the myopathic form in the practice of NBS in Japan.

Prior to proposing C12/C2 as the most promising NBS marker for CPT II deficiency, we investigated whether the four markers might give false-positive results in NBS for VLCAD deficiency that works next to CPT II. As shown in the scatter plots in Figure 1, the distributions of VLCAD deficiency cases overlap with those of CPT II deficiency cases across all four CPT II markers. In contrast, when C14:1—the characteristic marker for VLCAD deficiency—is plotted on the x-axis, the distributions of CPT II deficiency and VLCAD deficiency cases become clearly separated.

The distributional characteristics of the screening markers identified in this study provide valuable insights for marker selection and for the interpretation of false-positive results. CPT II deficiency has been classified as one of the long-chain fatty acid oxidation disorders. During the first decade of MS/MS-NBS, C16, C18:1 and (C16 + C18:1)/C2 in DBS were the primary markers for CPT II deficiency [12,13,14,15]. In 2011, target markers and cutoff ranges for various metabolic disorders in MS/MS-NBS were proposed as a result of “a worldwide collaborative project”, where C12 and C14 were listed together with the longer acylcarnitines (C16, C18, C18:1, C18:2), (C16 + C18:1)/C2, and low C0/(C16 + C18) [16]. Subsequently, several studies reported MS/MS-NBS data for CPT II deficiency that included C12 and C14 [17,18,19,20].

Concerning substrate specificity, CPT II was shown to be active with C8–C18 acyl-CoA [21]. However, minute relationship between chain length of fatty acids and dependency of their oxidation on carnitine shuttle had long been unclear. In 2023, Pereyra et al. showed insight into this question by experimental data on the ability of mice tissues to oxidize free fatty acids (FFA) of C6–C14 chain length without carnitine shuttle [22]. According to their findings, liver oxidized FFA of C6–C14, though the activity toward C12 and C14 were approximately 1/2 and 1/3 of the highest activity toward C8, respectively. Kidney oxidized FFA of C6–C10. Heart and skeletal muscle were unable to oxidize FFA of any chain length. In addition to the different biochemical mechanisms in various tissues, concentrations of C16-, C18- and C18:1-carnitine are far higher than those of the shorter fatty acylcarnitines in DBS while they show similar range of concentrations in serum [16], which appears to be due to relatively high content of C16-, C18- and C18:1-carnitine in blood cells. We speculate that complex contribution of these factors to the acylcarnitine profile in DBS should make C12-carnitine more sensitive and specific than the other candidates in the practice of MS/MS-NBS for CPT II deficiency in Japan.

The markers used in this study may also detect patients with carnitine–acylcarnitine translocase (CACT) deficiency or long-chain 3-hydroxyacyl-CoA dehydrogenase (LCHAD) deficiency. However, these conditions were not examined because they are even rarer in Japan than CPT II deficiency and sufficient data for evaluation are not available. If cases of these conditions are identified in the future, the performance of the markers evaluated in this study should be further validated.

## 5. Conclusions

The newly developed marker C12/C2 showed improved screening performance for CPT II deficiency in a highly imbalanced dataset. It appears to serve as a complementary or alternative marker in national NBS programs pending external validation. We are planning to conduct a prospective screening for CPT II deficiency to compare the performance of C12/C2 with the current markers (C16 + C18:1)/C2 and C14/C3.

## Figures and Tables

**Table 1:** Characteristics of the screening markers for CPT II deficiency.

	NBS-Positive Patients	NBS-Negative Patients with Myopathic Symptoms	Non-CPT II Deficiency
Total *n* (%)	21 (0.003%)	5 (0.001%)	683,391 (>99.9%)
C12/C0			
*n*	21	5	668,009
Mean (SD)	0.0376 (0.0227)	0.00880 (0.00291)	0.00355 (0.00178)
Median (Q1–Q3)	0.0277 (0.0186–0.0535)	0.00875 (0.00850–0.0102)	0.00320 (0.00245–0.00421)
Range	0.0147–0.0833	0.00432–0.0122	0.000040–0.216
C12/C2			
*n*	21	5	668,009
Mean (SD)	0.119 (0.0791)	0.0175 (0.0143)	0.00440 (0.00178)
Median (Q1–Q3)	0.0950 (0.0676–0.144)	0.0106 (0.00986–0.0207)	0.00413 (0.00324–0.00525)
Range	0.0338–0.343	0.00526–0.0409	0.000270–0.283
(C16 + C18:1)/C2			
*n*	21	5	600,090
Mean (SD)	1.84 (1.57)	0.240 (0.0738)	0.190 (0.0495)
Median (Q1–Q3)	1.14 (0.750–3.01)	0.220 (0.200–0.310)	0.184 (0.158–0.218)
Range	0.470–6.31	0.148–0.320	0.0150–1.85
C14/C3			
*n*	20	5	619,350
Mean (SD)	2.87 (2.40)	0.235 (0.0975)	0.119 (0.0453)
Median (Q1–Q3)	1.74 (1.00–4.41)	0.244 (0.220–0.268)	0.111 (0.090–0.140)
Range	0.690–8.39	0.0860–0.355	0.010–5.94

CPT II deficiency, carnitine palmitoyltransferase II deficiency; SD, standard deviation; Q1, the first quartile; Q3, the third quartile. There were missing values in the data of screening markers because the measured items varied among testing laboratories.

**Table 2:** Summary of performance metrics.

Indicators	C12/C0	C12/C2	(C16 + C18:1)/C2	C14/C3
Total, *n*	668,035	668,035	600,116	619,375
Number of patients, *n*	21	21	21	20
Cutoff value	0.0147	0.0333	0.470	0.675
Sensitivity (%)	100%	100%	100%	100%
Specificity (%)	99.8%	>99.9%	99.9%	>99.9%
PPV (%)	1.58%	51.2%	4.38%	30.8%
NPV (%)	100%	100%	100%	100%
Recall rate (%)	0.200%	0.006%	0.080%	0.010%
ROC-AUC (95% CI)	>0.999 (>0.999–>0.999)	1 (1–1)	>0.999 (>0.999–1)	1 (1–1)

95% CI, 95% confidence interval; NPV, negative predictive value; PPV, positive predictive value; ROC-AUC, the area under the receiver operating characteristic curve. The data are reported with three significant digits. The optimal cutoff value was determined by maximizing the Youden index.

## Data Availability

The data presented in this study are available on request from the corresponding author.

## Supplementary Materials

The following supporting information can be downloaded at: https://www.mdpi.com/article/10.3390/ijns12020036/s1, Figure S1: Receiver operating characteristic curves for the four screening markers for CPT II deficiency including myopathic form.
