# Supplementary material for: Comparison of Four Screening Markers [(C16 + C18:1)/C2, C14/C3, C12/C0, and C12/C2] for Carnitine Palmitoyltransferase II Deficiency in the Nationwide Newborn Screening Program in Japan
Source: Int J Neonatal Screen. 2026 May 15;12(2):36. doi: 10.3390/ijns12020036 (PMC13214869; doi:10.3390/ijns12020036)
Supplement: Supplementary file 1 [file IJNS-12-00036-s001.zip › IJNS-4138901-supplementary.pdf]

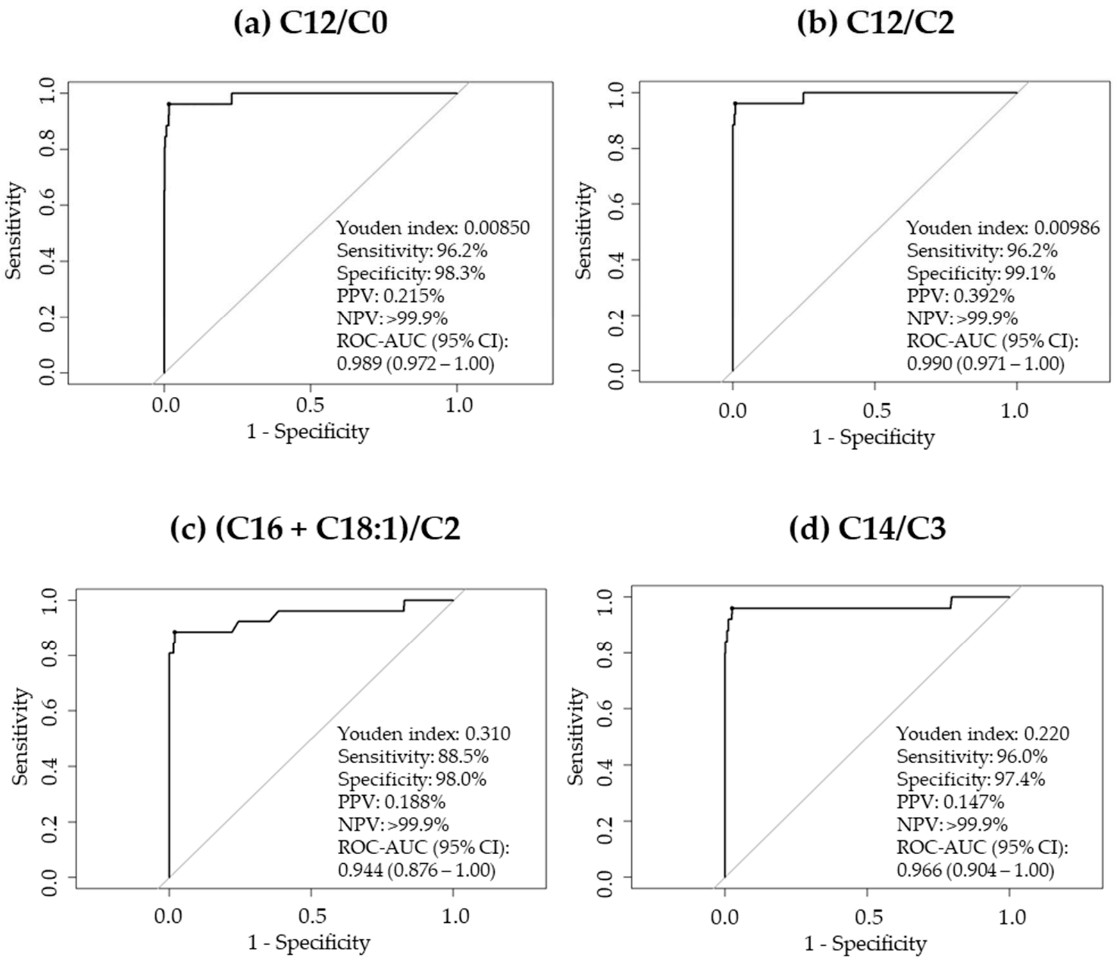

Figure S1. Receiver operating characteristic curves for the four screening markers for CPT II deficiency including myopathic form.

95% CI, 95% confidence interval; NPV, negative predictive value; PPV, positive predictive value; ROC-AUC, area under the receiver operating characteristic curve; PR-AUC, area under the precision–recall curve. The total numbers of samples analyzed for C12/C0, C12/C2, (C16 + C18:1)/C2, and C14/C3 were 668,035, 668,035, 600,116, and 619,375, respectively, and the numbers of patients with CPT II deficiency were 26, 26, 26, and 25, respectively. Data are reported to three significant digits.
